# Wearable Epileptic Seizure Prediction System with Machine-Learning-Based Anomaly Detection of Heart Rate Variability

**DOI:** 10.3390/s20143987

**Published:** 2020-07-17

**Authors:** Toshitaka Yamakawa, Miho Miyajima, Koichi Fujiwara, Manabu Kano, Yoko Suzuki, Yutaka Watanabe, Satsuki Watanabe, Tohru Hoshida, Motoki Inaji, Taketoshi Maehara

**Affiliations:** 1 Division of Informatics and Energy, Faculty of Advanced Science and Technology, Kumamoto University, Kumamoto 860-8555, Japan; 2 Fuzzy Logic Systems Institute, Iizuka 820-0067, Japan; 3 Section of Liaison Psychiatry and Palliative Medicine, Graduate School of Medical and Dental Sciences, Tokyo Medical and Dental University, Tokyo 113-8510, Japan; miholppm@tmd.ac.jp (M.M.); yoko.12.suzu@gmail.com (Y.S.); 4 Graduate School of Engineering, Nagoya University, Nagoya 464-8603, Japan; fujiwara.koichi@hps.material.nagoya-u.ac.jp; 5 Graduate School of Informatics, Kyoto University, Kyoto 606-8501, Japan; manabu@human.sys.i.kyoto-u.ac.jp; 6 Amekudai Hospital, Naha 900-0005, Japan; wata2yt@gmail.com; 7 Department of Psychiatry, National Center Hospital of Neurology and Psychiatry, Kodaira 187-8553, Japan; satsuki218@gmail.com; 8 Department of Psychiatry, Saitama Medical University Hospital, Saitama 350-0495, Japan; 9 National Hospital Organization Nara Medical Center, Nara 619-1124, Japan; t-hoshida@takanohara-ch.or.jp; 10 Department of Neurosurgery, Tokyo Medical and Dental University, Tokyo 113-8510, Japan; inamnsrg@tmd.ac.jp (M.I.); maehara.nsrg@tmd.ac.jp (T.M.)

**Keywords:** epilepsy, electrocardiography, heart rate variability, multivariate statistical process control, wearable system, machine learning, seizure prediction

## Abstract

A warning prior to seizure onset can help improve the quality of life for epilepsy patients. The feasibility of a wearable system for predicting epileptic seizures using anomaly detection based on machine learning is evaluated. An original telemeter is developed for continuous measurement of R-R intervals derived from an electrocardiogram. A bespoke smartphone app calculates the indices of heart rate variability in real time from the R-R intervals, and the indices are monitored using multivariate statistical process control by the smartphone app. The proposed system was evaluated on seven epilepsy patients. The accuracy and reliability of the R-R interval measurement, which was examined in comparison with the reference electrocardiogram, showed sufficient performance for heart rate variability analysis. The results obtained using the proposed system were compared with those obtained using the existing video and electroencephalogram assessments; it was noted that the proposed method has a sensitivity of 85.7% in detecting heart rate variability change prior to seizures. The false positive rate of 0.62 times/h was not significantly different from the healthy controls. The prediction performance and practical advantages of portability and real-time operation are demonstrated in this study.

## 1. Introduction

Epilepsy is a chronic neurological disorder characterized by recurrent seizures that affects approximately 1% of the world’s population [1]. Antiepileptic drugs are ineffective in controlling seizures in up to 30% of patients to whom it is administered, who can develop intractable epilepsy with the administration of more than three antiepileptic drugs [2].

Convulsions or loss of consciousness associated with uncontrolled seizures may cause serious injuries to the patients themselves and the people around them. Early detection of epileptic seizures prior to symptoms allows for early intervention with fast-acting medication, vagus nerve stimulation, or evasive action against accidents. A questionnaire answered by 141 epilepsy patients showed that more than 90% of the patients desired seizure prediction [3].

Although epileptic seizure prediction using electroencephalogram (EEG) data has achieved reliable sensitivity and specificity [4,5,6,7], intolerance to motion artifacts with scalp EEG and craniotomy techniques required for invasive EEG would be a major barrier to dissemination. In addition, because the real-time analysis of the EEG channels acquired at a sampling rate higher than 500 Hz requires huge machine power for calculation, the current application software achieving standalone seizure prediction can hardly be implemented on a handheld device, such as a smartphone.

As epileptic seizures affect the autonomic nervous function, peri-ictal changes are seen in the heart rate and electrocardiogram (ECG) data [8,9,10]. The parasympathetic and sympathetic outputs to the heart are modulated by the central autonomic network, which includes the insular cortex, orbitofrontal cortex, cingulate, amygdala, hypothalamus, and periaqueductal gray matter. Excessive neural activation associated with epileptic seizures distributed in these brain regions affects the functioning of the central autonomic network, which is reflected by heart rate variability. Hence, the practicality of prediction care can be improved by monitoring the cardiac autonomic changes in the early ictal phase [11,12,13] and preictal phase [14] by heart rate variability (HRV) analysis [15,16] with a portable Holter monitor. A systematic review of HRV and epileptic seizures was performed, which showed that the HRV and heart rate change before seizure onset [17]. Feature extraction from HRV indices preceding seizure onset using the machine learning technique should contribute toward realizing automatic seizure prediction, whereas the Lorenz-plot-based method predicted seizures in only three of the seventeen patients [18]. Although time-frequency analysis [19], nonlinear analysis of HRV using fuzzy clustering [20], and the machine learning method based on support vector machine [21] demonstrated greater prediction sensitivity than the random predictor in retrospective analysis of preictal ECG collected from video-EEG recording, the feasibility of real-time analysis and prospective clinical applications has not been examined. Evaluation of the real-time capability through ECG data acquisition, calculation of HRV indices, and calculation of analytic features operating on a wearable system is crucial for demonstrating the feasibility of the prediction system, because seizure warnings would help avoid seizure-related accidents, improve patient quality of life, and provide novel treatment strategies, particularly for intractable epilepsy.

To improve the positive predictive value in the seizure prediction, our group reported a novel analytical method using machine learning. The method employs multivariate statistical process control (MSPC) [22] for anomaly detection to identify minority samples that have different characteristics to the remaining samples.

In this study, the feasibility and reliability of seizure prediction as a diagnostic tool that is practical for daily use by patients is evaluated with a prototype in hospitals during long-term video-EEG monitoring, which is categorized as the phase 2 validation framework [23]. Section 2 describes the bespoke wearable system for HRV measurement and analysis for seizure prediction. The experimental methods for evaluating the seizure prediction capability are explained in Section 3 and the results are shown in Section 4. Section 5 discusses the results from instrumentation engineering and biomedical significance perspectives. Section 6 concludes the study.

## 2. Epileptic Seizure Prediction System

### 2.1. System Composition

**Figure 1:**
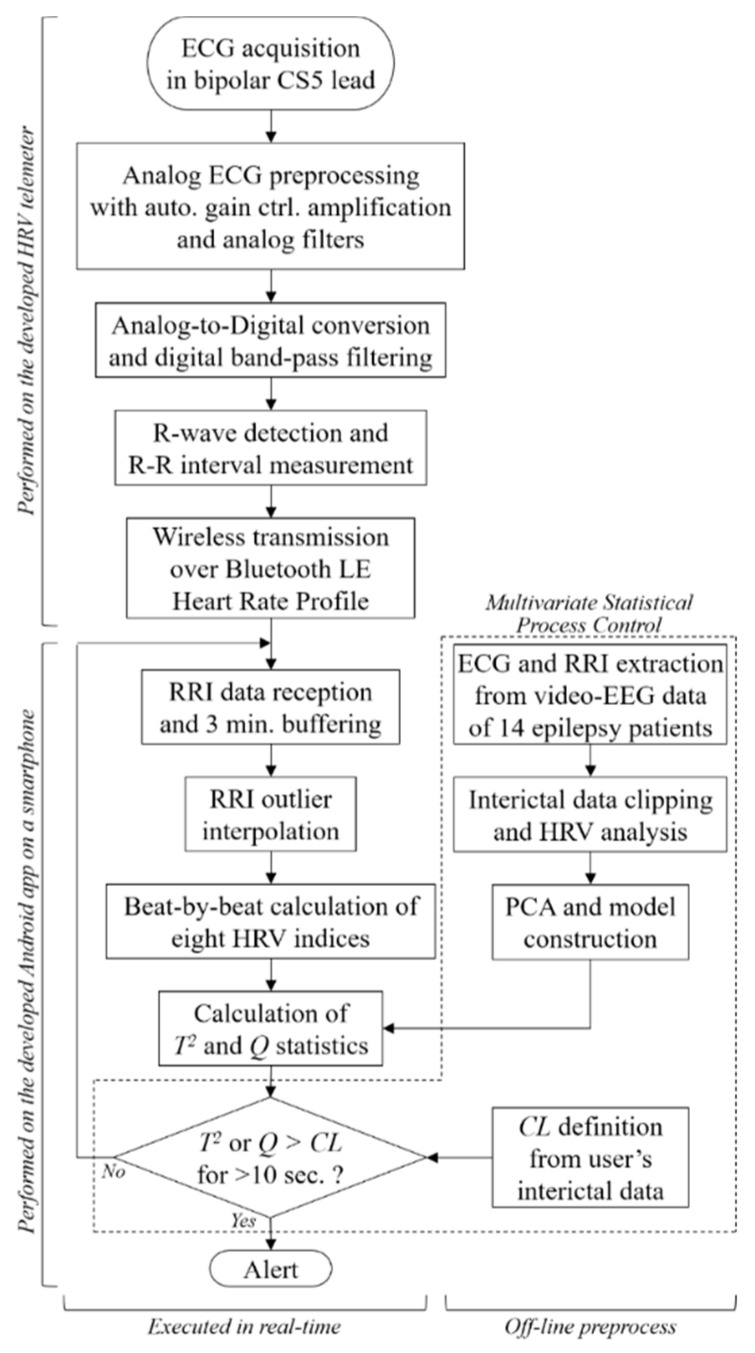
System overview and signal processing workflow of the proposed epileptic seizure prediction system.

**Figure 2:**
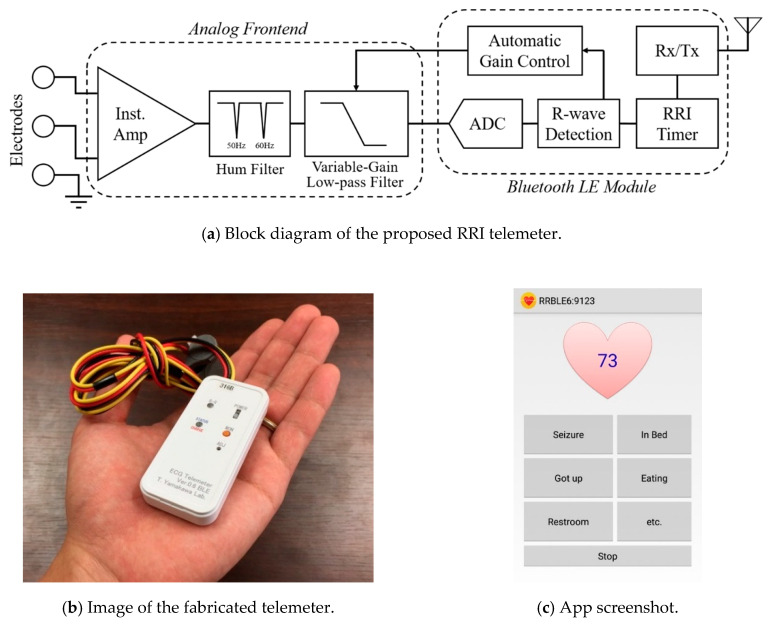
(**a**) Block diagram and (**b**) image of the proposed RRI telemeter. (**c**) Screenshot of the developed Android app showing memo buttons; current heart rate appears on the heart while monitoring. Instrumentation amplifier (Inst. Amp), analog-to-digital converter (ADC), receiver and transmitter (Rx/Tx).

Figure 1 shows an overview and a flowchart of the proposed seizure prediction system. The developed wearable system consists of an R-R interval (RRI, the time elapsed between two successive R-waves of the QRS signal on the electrocardiogram) telemeter and a smartphone. Although many wearable heart rate monitors are available on the market, the provided functions have limitations for use in the proposed system, which requires a sampling rate higher than 500 Hz for HRV analysis, device-embedded real-time RRI calculation to reduce the calculation load of the smartphone, and an automated gain control function to be operated by a nonspecialized user. Thus, we developed an original telemeter to realize the functions shown in the upper half of Figure 1 within a small and lightweight form factor. Figure 2a shows the block diagram of the telemeter that measures the RRIs of the ECG. The device has small dimensions of 74 × 34 mm, with a thickness of 12 mm, excluding the three one-meter ECG lead wires, as shown in Figure 2b, and a long battery life for wearable healthcare applications. The analog front-end circuitry of the telemeter amplifies the AC-coupled ECG signal obtained from the three disposable electrodes using an instrumentation amplifier, and reduces the noise using a 50/60 Hz notch filter and a variable gain low-pass filter. ECG signals preprocessed in the analog front end are sampled by a 1 kHz 10-bit analog-to-digital converter and analyzed using threshold-based R-wave detection [24] in a cortex-M0 microcontroller built into a Bluetooth module (EYSFCNZXX, Taiyo Yuden, Japan). The sampling frequency was compatible with the 1 kHz sampled video-EEG data used for the phase 1 validation of our previous method [22]. In a simple threshold discrimination, the low-frequency baseline drift and P- and T-waves (which have the same polarity as R-waves in the Standard Lead II configuration) of ECG signals may lead to false positives of R-wave detection. Accordingly, a quasi-band-pass filtering program determines the pulse width of the digitized ECG and forwards the digitized ECG to a comparator when the pulse width is within 3–50 ms. A timer counts and transmits RRIs to the smartphone via the Bluetooth 4.0-LE wireless connection, which operates under the smartphone’s heart rate profile. Here, the RRIs for 60 beats are stored in the telemeter’s memory to avoid data loss due to transmission errors in the wireless connection. An automatic gain control function programmed in the telemeter microcontroller enables easy operation, which can be performed by nonspecialized subjects with simple instructions [25,26].

### 2.2. HRV Analysis

The bespoke smartphone software receives and analyzes RRI data in real time (Figure 2c) to derive HRV indices of autonomic nervous activities. The HRV indices used for the epileptic seizure prediction consist of four time-domain indices (mean of RRI (meanNN), standard deviation of the RRI (SDNN), root mean square of the differences of adjacent RRIs (RMSSD), and the number of pairs of adjacent RRIs whose difference is greater than 50 ms within a given length of measurement time (NN50)) and four frequency-domain indices (total power (TP), variance of RRI, power of the low-frequency band of 0.04–0.15 Hz (LF), power of the high-frequency band of 0.15–0.4 Hz (HF), and ratio of LF to HF (LF/HF)) [15].

HRV indices are derived from statistical or frequency analysis of a three-minute moving window, so an outlier contamination in the RRIs would adversely affect the indices for the following three minutes. Hence, the outlier RRIs should be replaced using the following real-time compensation techniques, which were implemented as a function on the smartphone app in this work.

The observed RRIs for 180 s are stored in a first-in, first-out buffer. The median absolute deviation *RRI_MAD_* of the *i*-th RRI, i.e., *RRI_i_*, is calculated with the stored RRIs (*RRI_n_* | *n*: 1~i-1) as

(1) $$RRI_{MAD}=median|RRI_{i}-median\;RRI_{n}|$$

where the function “median” gives the median of the input variables. The normal RRI range is

(2) $$median\;RRI_{n}-4\sigma <RRI_{i}<median\;RRI_{n}+4\sigma$$

where the standard deviation of the RRI is $\sigma =1.4826\times RRI_{MAD}$.

The RRI outliers, which are less than the lower limit, are removed from the RRI data used for HRV analysis. However, when the observed RRI is greater than the upper limit, it is assumed that multiple heartbeats have been missed. Accordingly, the number of missed heartbeats *N* is estimated as

(3) $$N=round\;\left(\frac{RRI_{i}}{median\;RRI_{n}}\right)$$

where the function “round” returns the rounded value of the input value and the outlier is replaced with the *N*-th corrected RRIs by

(4) $$\hat{RRI}=\frac{RRI_{i}}{N}$$

The time-domain indices are calculated from the buffered RRIs. For the frequency-domain indices, the stored RRIs are resampled to ensure uniform intervals for the frequency analysis. The third-order spline is used for the RRI interpolation and the sampling rate is 4 Hz. The power spectrum density of the resampled RRI data is calculated using an autoregressive model of the order of 40 and the frequency-domain indices are derived from the power spectrum density according to Fujiwara et al. [22].

### 2.3. Anomaly Detection Prior to Epileptic Seizure

Univariate statistical process control monitors each variable with upper and lower limits, which is a simple approach to anomaly detection. However, univariate statistical process control is unsuitable for anomaly detection because it cannot monitor changes in correlated variables. To consider the correlation between the HRV indices, we employed MSPC, which is a machine learning technique that has been applied to anomaly detection in multivariate systems [27,28,29].

In MSPC, principal component analysis models linear correlations, referred to as principal components. In this study, six principal components with the highest contribution rates describe major trends in the changes of the HRV indices.

MSPC defines the normal operating conditions as two indices, namely the *Q* and *T*^2^ statistics [30], which monitor the gap between sample and model data. The *Q* statistic is the squared distance between a sample and the subspace spanned by the principal components; that is, the *Q* statistic measures the dissimilarity between the sample and the model data from the viewpoint of the correlation between the variables. The *T*^2^ statistic is the Mahalanobis distance between a sample and the origin in the subspace spanned by the principal components. When the *T*^2^ statistic is low, the sample is close to the mean of the model data. The statistics are obtained by the following determinants

(5) $$Q=x^{T}\left(I-V_{R}V_{R}^{T}\right)x$$

(6) $$T^{2}=x^{T}V_{R}{Ó}_{R}^{-2}V_{R}^{T}x$$

where *x* denotes the column vector consisting of the eight sampled HRV indices. Here, $V_{R}\in R^{8\times 6}$ is a loading matrix, the *r*-th column represents the direction of the *r*-th principal component; ${Ó}_{R}\in R^{6\times 6}$ is a diagonal matrix, where the diagonal elements are standard deviations of the corresponding principal components; and *I* is the identity matrix. Equations (5) and (6) are not computationally expensive, so MSPC is suitable for real-time monitoring of HRV. There are few instances of epileptic seizures (i.e., anomalies) that are collected in data, so MSPC models the normal data. The collected RRI data and calculated parameters are analyzed for seizure prediction according to Algorithm 1.
**Algorithm 1** Seizure prediction algorithm1**set***τ* [0] ← 0, *C* [0] ← Ɲ.2**while do**3Collect the newly measured *t*-th RRI *y*[t].4Extract and preprocess the HRV indices *x*[t].5Calculate the *t*-th *T*^2^ [*t*] and *Q*[*t*] from *x*[*t*] by using Equations (5) and (6).6**if**$\left\{\left(T^{2}\left[t\right]\;⋁\;Q\left[t\right]>CL\right)\;AND\;C\left[t-1\right]=Ɲ\right\}\;OR$

$\left\{\left(T^{2}\left[t\right]\;⋁\;Q\left[t\right]\leq CL\right)\;AND\;C\left[t-1\right]=Ƥ\right\}$7**then**τ[t]=τ[t−1]+y[t]8**else**τ[t]=09**end if**10**if**τ[t]≥10 s11**then**C[t]=¬C[t−1] AND τ[t]=012**end if**13Wait until the next RRI data *y* [*t* + 1] are measured.14**end while**

In this algorithm, *y*[*t*] denotes the *t*-th RRI and *t* denotes the number of samples from the start of the analysis. Here, *τ* is a time counter variable and *C* is a binary indicator (Ɲ or Ƥ, ¬Ɲ = Ƥ) of the patient status, where Ɲ and Ƥ indicate “interictal” and “preictal,” respectively. To ignore instantaneous HRV changes such as those due to rapid motion, the patient status is determined as preictal when either the *T*^2^ or *Q* statistic exceeds its control limit for more than ten seconds continuously.

The control limits (CLs) of the *Q* and *T*^2^ statistics are defined as *α*% confidence limits. CLs are determined by the lower *α*% and upper *α*% of the confidence interval for samples representing the normal condition. The sensitivity and the specificity of the MSPC are controlled by *α*, which was set to 99%.

The analysis method mentioned above was implemented on an Android app programmed using the Java programing language. In this study, the modeling parameters of *V_R_* and *Σ_R_* were used from Fujiwara et al. [22].

## 3. Experimental Methods

### 3.1. MSPC Model Construction

The interictal HRV data for constructing the MSPC model were collected from refractory epilepsy patients who were admitted to three departments in Japan: the Department of Neurosurgery, Medical Hospital, Tokyo Medical and Dental University (TMDU); the Department of Psychiatry, National Center Hospital of Neurology and Psychiatry (NCNP); and the Department of Epileptology, Tohoku University Hospital (TUH). Patients underwent clinical video-EEG monitoring for presurgical evaluation or seizure assessment. Data from fourteen patients collected by Fujiwara et al. (2016) were used with the approval of the Medical Research Ethics Committees from all participating departments. Two or more experts from the Japan Epilepsy Society labeled seizures and epileptic interictal spikes from video-EEG data of the awake state to allow creation of a dataset containing only normal data. The HRV indices were calculated from the cropped ECG and the learning procedure of the MSPC model was performed by MATLAB according to (Novak et al., 1999). The MSPC does not model abnormality (i.e., seizures), and collecting clean ECG data during a seizure is difficult because of the contamination of seizure motion artifacts and the low frequency of seizures.

### 3.2. Measurement Setup and Protocols

The prospective study was conducted on epilepsy patients admitted to TMDU, NCNP, and the Department of Psychiatry of Nara Medical Center (NMC), Japan, for clinical video-EEG monitoring. The experiments were approved by the Medical Research Ethics Committees of TMDU, NCNP, and NMC, and written informed consent was obtained from each participant.

The subjects rested in a supine or sitting position in a bed inside a monitoring room and their prescribed antiepileptic drugs were reduced from the usual dosage, according to the standard procedure of video-EEG monitoring. The video, ECG, and EEG data were recorded simultaneously using a long-term video-EEG monitoring system (Neurofax EEG-1200, NIHON KOHDEN, Japan) with sampling frequencies of 500 or 1000 Hz for both ECG and EEG.

The fabricated wearable RRI telemeter, which has disposable ECG electrodes and a bipolar CS5 lead configuration, was used simultaneously with the video-EEG monitoring system. Data were collected with an Android smartphone (Blade S Lite g02, ZTE Japan K. K., Japan) fixed to the bedside and tethered for continuous power. The internal clock of the smartphone was manually synchronized with the clock of the video-EEG monitoring system, because the network security regulations do not permit use of the network time protocol. The recording with the wearable system was continued as long as possible during the video-EEG monitoring.

The CLs were calculated during post-processing after video-EEG monitoring, because the interictal periods must be determined from the video-EEG data diagnosed by clinical specialists. Therefore, only steps 6–12 in Algorithm 1 were executed on the PC after the experiments.

The system was evaluated with video-EEG monitoring, which is considered the gold standard for commercial seizure detection devices [31,32,33]. However, video-EEG monitoring restricts body motion and autonomic change from normal daily life, which may lead to the underestimation of the number of false positives. Therefore, in addition to the recommended validation methods [34], the system was evaluated on the healthy subjects (controlled to be consistent in gender and age) and focused on false positives from the uncontrolled situation of their daily life. The healthy subjects without a history of cardiac and neuronal diseases were asked to wear the system during their daily life, except while sleeping. The same criteria for data exclusion with the patient data was used.

## 4. Results

### 4.1. Patient Attribution

Eighteen focal epilepsy patients were admitted to the study who were not involved previously. Data from two patients were removed because they did not have a seizure. The data obtained during the nocturnal period between 20:00 and 08:00 and during naps (observed in video) were removed from the assessment, as the risk of accidental injury due to focal seizures during sleep is significantly lower than those during awake periods. Moreover, the HRV is affected by micro-arousal and sleep stage transitions [35,36], which may increase false positives. Thus, nine patients were excluded as they had seizures only during sleep. Table 1 and Table 2 detail the remaining seven patients and their analyzed episodes, respectively. Premature ventricular contraction may also cause false positives.

To independently evaluate the performance, periods of data affected by such pathophysiologic contaminants were identified by cardiovascular specialists certified by the Japanese Circulation Society and were removed from the evaluation. Additionally, contaminated motion artifacts in the EEG traces increase the difficulty to accurately determine seizure onsets and interictal discharges. These noisy intervals of EEG data are defined with the analyzed results, such as the anomalies in the HRV indices, *Q/T*^2^ statistics, and RRI interpolation performed by Equations (1)–(4), then removed from the validation of the seizure prediction performance under visual inspection performed by two or more specialists certified by the Japan Epilepsy Society. The remaining data were reviewed and diagnosed by two or more specialists certified by the Japan Epilepsy Society and the start of the seizure symptoms was labeled as the seizure onset. On the other hand, the time intervals greater than 30 min from seizure onsets, sleep activity, and interictal discharges were labeled as the interictal periods by the specialists observing the video and EEG. The interictal data were used to evaluate the false positives due to the malfunction of the proposed method; they were independent from the pathological physiologic changes in the patients.

In addition, the data from seven healthy controls whose age and gender were balanced with the patients were collected with the proposed system during their daily life, as shown in Table 2.

### 4.2. Measurement Accuracy and Reliability

**Figure 3:**
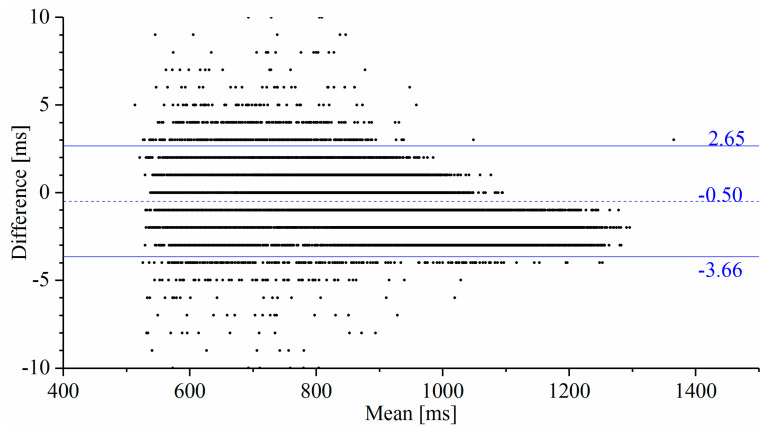
Bland–Altman plot comparing the RRIs obtained from the reference ECG and the proposed system. The measurement bias indicated by the dashed-line and the stochastic error range indicated by the solid lines defined by the limits of agreement are sufficiently low for HRV analysis. No significant proportional bias is observed in the result of the regression analysis of this plot.

The interictal RRIs obtained using the proposed system and from the video-EEG monitoring system were compared using Bland–Altman analysis [37]. The RRIs were derived from the ECG channel recorded on the video-EEG data with the same MATLAB features used in [22]. These RRIs were aligned with those collected by the developed system to be on the same timeline in order to calculate the statistical indices of the Bland–Altman analysis. The Bland–Altman plot (Figure 3) describes the compatibility of the two compared systems. The horizontal and vertical axes represent the mean and the difference (subtracting the device RRI from the reference RRI) of the RRIs, respectively, obtained from the compared systems. The dashed horizontal line and the adjoining number indicate the mean of the difference and represent the measurement bias of the proposed system against the reference system. The two solid horizontal lines represent the intervals of the limits of agreement of the measurement difference from the Bland–Altman analysis.

Table 3 lists the measurement failure rates of the RRIs calculated from the number of RRI outliers beyond the range defined by Equation (2). Here, the RRI outliers have been replaced using the outlier modification method (from Section 2.2), so that the effects of the outliers on the HRV indices are removed to avoid inducing false positives.

### 4.3. Seizure Prediction

Seizure prediction between 15 min prior to seizure onset and the moment immediately before seizure onset was considered to be successful [22], i.e., the prediction horizon was 15 min. Table 4 lists the durations when the *Q* and *T*^2^ statistics exceed the control limit for more than 10 s, where the seizure starts at *t* = 0.

**Figure 4:**
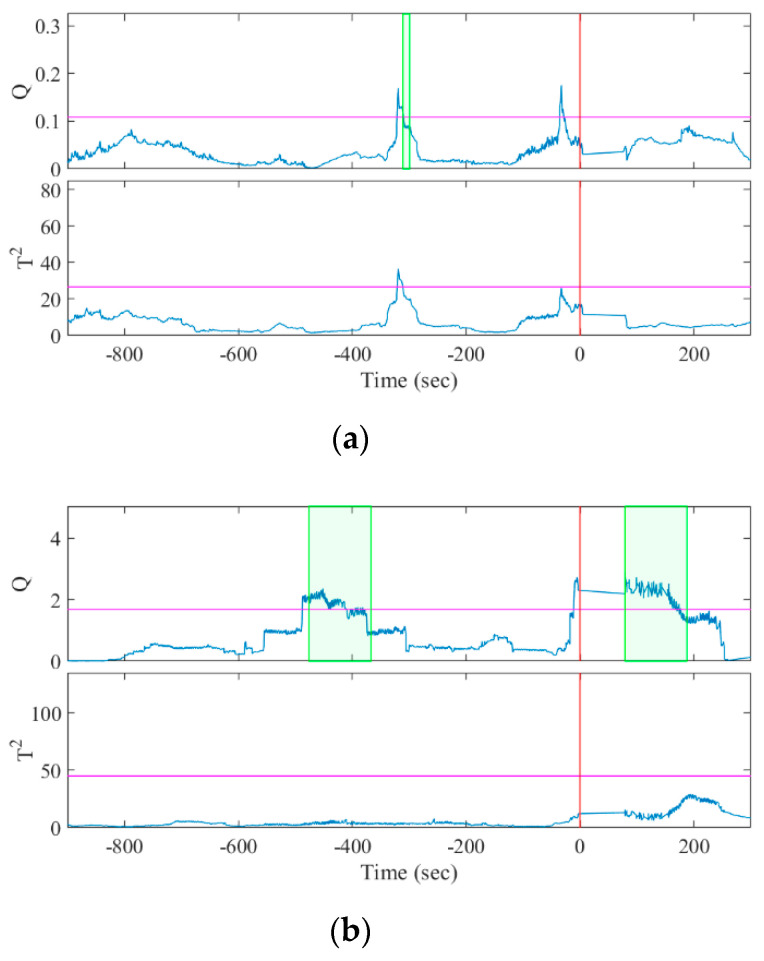
Results of MSPC analysis for seizure episodes (**a**) A1 and (**b**) B1. The horizontal lines and vertical lines indicate the control limits and the seizure onset, respectively. The colored bands denote C[t]=Ƥ, indicating the discriminated preictal change.

**Figure 5:**
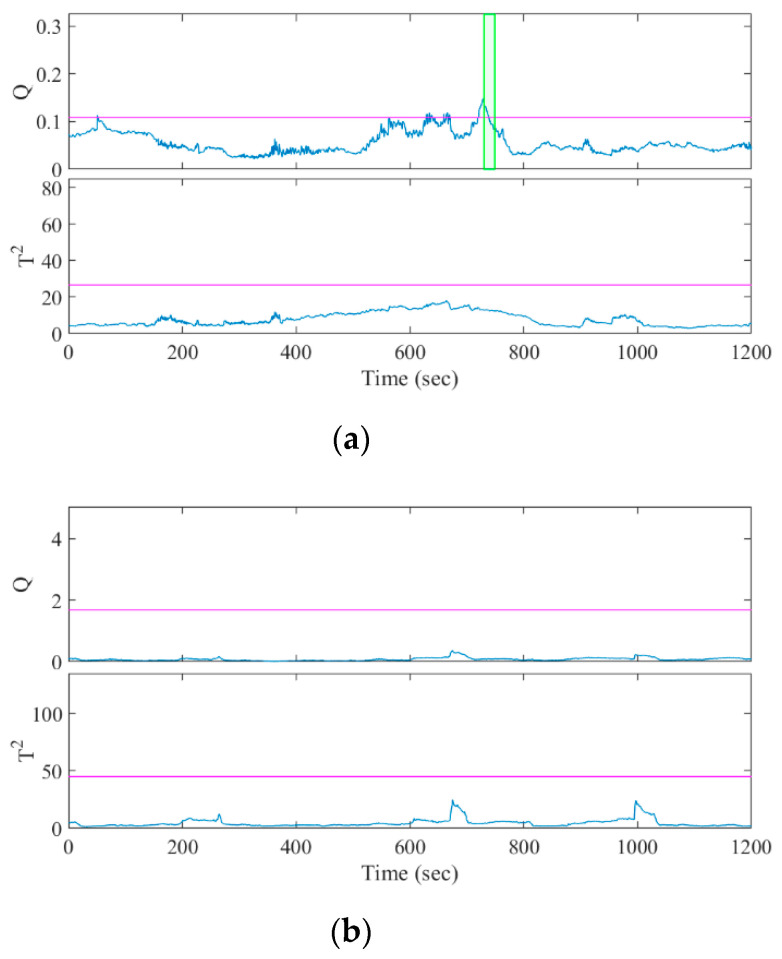
Result of MSPC analysis for the interictal episodes of (**a**) patient A, selected to include false positives; and (**b**) patient B, without the false positives.

The seizures were identified by a character for the patient identity and a number for the seizure instance, e.g., A1 was patient A’s first seizure. Twelve of the fourteen seizures were predicted correctly with a sensitivity of 85.7% by the *Q* statistic and two of fourteen seizures were predicted correctly with a sensitivity of 14.3% by the *T*^2^ statistic. The one-sided sign test [38,39] of the *Q* and *T*^2^ statistics showed that the sensitivity of our algorithm was significantly greater than and lower than the chance level, respectively (both *p* = 0.0065). Figure 4 and Figure 5 show examples of how the *Q* and *T*^2^ statistics change before a seizure onset and during the interictal period, respectively. The colored band in the figures highlights the duration when C[t]=Ƥ was satisfied, indicating that the statistic exceeded CL (represented with the colored horizontal line) by 10 s and was discriminated as preictal in Algorithm 1.

The false positives during the interictal periods were analyzed and summarized for each patient in Table 5 using a popular method in the field of seizure detection systems [40]. The false positive rate is defined as the number of false positives per hour.

## 5. Discussion

EEG is the most popular biomarker for seizure prediction, but HRV is rarely used [41], although the effects of epileptic seizures on the cardiac autonomic function have been reported in the early ictal phase [11,12,13] and the preictal phase [14]. Previous approaches of seizure prediction achieved comparable performance with HRV and time-frequency analysis [19], fuzzy clustering [20], and a support vector machine classifier [21]. However, only retrospective analysis of video-EEG data was performed, and real-time analyses are not reported. In this study, a wearable system was used with video-EEG monitoring. The HRV indices were calculated in real time on a smartphone that contained a precalibrated MSPC model. Only the control limit, which is a discrimination threshold of seizure prediction, was calculated after measurement, since the interictal periods should be defined by specialists through a diagnosis of the video-EEG data. In clinical situations, the proposed system would be used after a definitive diagnosis has been made with video-EEG monitoring, with the control limits being calculated prior to use. In addition to prediction, the smartphone app sends data to a server, so remote monitoring in telemedicine may enable rapid fine-tuning of CLs and *τ* via the network. Thus, the proposed system demonstrated adequate real-time HRV monitoring and seizure prediction.

The small size of the proposed system, consisting of a telemeter and a smartphone, allows patients to hold the system. The battery life depends on the smartphone model and the telemeter’s operating duration of more than 48 h after a full charge. Considering that a fully charged Android smartphone depletes its 3010 mAh battery in 14.12 ± 0.68 h (mean ± SD, *n* = 6) under continuous operation, the proposed wearable system has sufficient battery life for daily activity monitoring from 09:00 to 18:00. Although the fabricated telemeters failed to measure the RRIs of 3.46 ± 1.91% (mean ± SD) in the interictal episodes, the failure rates are sufficiently small to be used for HRV analysis, because an RRI failure that is less than 5% does not cause a significant error in the HRV indices when they are compensated properly [16,42]. Meanwhile, the effect of the RRI outliers can be minimized through RRI outlier compensation (Section 2.2). The intersubject variance in the RRI failure rates suggests that it was affected by the stability of the electrodes and patients, because the threshold-based R-wave detection method adopted in the proposed telemeter is relatively weak against the baseline drift of ECG. Since the present study showed that the original telemeter exhibited sufficient performance only in situations where patient activity was limited and the false positive rates in healthy controls were relatively higher than that in patients, device lead-off and motion artifacts in daily life activity should be discussed in the upcoming phase 3 and 4 studies. Garment-type ECG electrodes could be a solution for improving usability and reducing motion artifacts originating from multiplied wire leads [43].

Although the numbers of seizures and patients used for evaluation are relatively small in comparison to the retrospective studies for seizure prediction [44], the number of patients used for the learning dataset is comparable because the MSPC model was constructed from fourteen subjects in our previous study [22]. In general, the number of events per variable is recommended to be as least ten [45] when a statistical model is built. The proposed MSPC model has eight input HRV features that were calculated from an RRI between two heartbeats. The model was constructed with 18.9 h of interictal data that included more than ten thousand heartbeats [22]. Thus, the sample size for model construction was appropriate for this study. Meanwhile, statistical significance was shown for the prediction sensitivity, because fourteen seizures were obtained from seven patients. A study with a larger number of subjects with controlled syndromes and focus locations may help in understanding the variance in prediction latency.

In Fujiwara et al. [22], epileptic seizure prediction with a sensitivity of 91% and a false positive rate of 0.7 times/h was performed using an offline retrospective analysis of the ECG extracted from long-term video-EEG monitoring. In this current study, the measurement and analysis functions were implemented in the wearable system and operated in real time. The developed system realized a sensitivity of 85.7% and a false positive rate of 0.62 times/h, where the MSPC model was constructed with previously unseen data (i.e., different patients) and the *Q* statistics were used independently for seizure prediction. The proposed system’s sensitivity is comparable to previous systems (78–100%) introduced in the review papers, which summarized 23 seizure prediction methods [40,46]; however, most of them did not consider wearability or real-time analysis capability, so are not considered suitable for practical use. Conversely, the retrospective studies of EEG-based seizure prediction achieved a false positive rate within the range of 0.06–0.39 times/h. The clinical studies of the seizure detection (not prediction) system [33,47] were comparable within the range of 0.04–0.16 times/h. In recent retrospective studies on seizure prediction using HRV analysis, Pavei et al. [48] presented an epileptic seizure prediction algorithm adopting an SVM classifier for HRV signals that forecasted seizures with a sensitivity of 94.1% and a false positive rate of 0.49 in patients with epilepsy and 0.19 in healthy subjects. Billeci et al. [21] proposed an HRV-based patient-specific seizure prediction method using recurrence quantification analysis, with an average sensitivity of 89.6 and a false positive rate of 0.41 per hour. The interview-based patient survey [3] showed that patients require high sensitivity from seizure prediction devices, whereas they regard specificity as secondary. However, the majority of patients required false alarms to be less than the correct predictions. Therefore, the present method requires further development to reduce the false positive rate.

There was no significant reduction in the false positive rate from our previous study. The results support our previous study’s hypothesis that the false positives may be due to body motion affecting the autonomic nervous system and HRV. As the patients rested in a sitting or supine position for most of the time during the video-EEG monitoring, the probability of body motion occurrence in the present study is considered similar to the previous study. The calculation of the t-test showed that the difference in the false positive rate of *Q* and *T*^2^ statistics between the patient and healthy groups was not statistically significant (*p* > 0.05). These results suggest that the autonomic activity rates of the healthy subjects and the epilepsy patients are not significantly different during interictal periods, where there is no effect from the interictal discharges.

The false positive rate of the *Q* statistic was significantly high in patient A. The data length of the extracted interictal periods of patient A was shorter than other patients because the patient had more seizures and interictal discharges than others. The insufficient length of the interictal data made the control limit unreliable. In patient C, two of the four false positives occurred while eating in the *Q* statistic, while three of the four false positives in *T*^2^ occurred while eating. Patients A and C had temporal lobe epilepsy (TLE), the severity of which may be related to abnormal heart rate regulation [49,50]. Considering preictal episodes A3 and C2, which were not predicted by the *Q* statistic, the interictal period should be carefully selected for appropriate control limit definition. However, the extracted interictal episodes were shorter than those in previous studies [5], because the severe exclusion criteria were defined to strictly exclude the epileptiform activity from the learning and CL tuning dataset [22]. Further work is required to determine sufficient durations of the interictal episodes for tuning of CLs and to develop a MSPC model specific to TLE patients.

Patient F showed a significantly high false positive rate in the *T*^2^ statistic. According to the results of the video-EEG assessment, four of thirteen false positives occurred during a cognitive function test and seven occurred when the patients were focused on their hobbies (e.g., making plastic models), which suggests that HRV changes occurred due to the mental workload, as seen from the *T*^2^ statistic. This hypothesis coincides with our previous results, which showed that the *T*^2^ statistic exerted a better discrimination performance than the *Q* statistic in preventing drowsy driving accidents using HRV analysis and MSPC [51].

This study has several limitations that should be focused on in future work. Only Japanese patients with limited epilepsy syndrome (focal epilepsy) were selected, which should be addressed in future work. The developed system was examined under limited conditions at the hospital, as precise evaluation of the prediction sensitivity requires the determination of accurate seizure onset and because the false positive rate requires reliable evidence of isolation from the effect of interictal discharges or sleep. Hence, we selected this limited situation that could facilitate simultaneous video-EEG monitoring, and the results were compared with the healthy controls in unlimited situations. However, evaluation of the proposed system considering practical situations in a patient’s daily life will be performed in future work to demonstrate the efficacy of a wearable seizure alert system. The ictal and interictal data while sleeping were excluded from the analysis. As suggested in the clinical trial in a seizure detection device [33], the development of an alternative MSPC model constructed only from the interictal sleep data may have the ability to predict sleep seizures, which remains to be developed and evaluated in future work. In addition, the intervals that were difficult for labeling preictal or interictal phases due to artifact contamination in the EEG data were removed from analysis retrospectively, although this offline preprocessing cannot be achieved in real-world situations. This may lead to the underestimation of the number of false positives and should be addressed in the upcoming phase 3 trial.

## 6. Conclusions

A prototype of a wearable system for epileptic seizure prediction was developed in this study. A bespoke telemeter operated by a nonspecialized person demonstrated sufficient accuracy and reliability for RRI measurement in comparison with the reference ECG of the video-EEG monitoring system. The *Q* and *T*^2^ statistics for the MSPC model were computed in real time and there was a sensitivity of 85.7% with a false positive rate of 0.62 times/h for the *Q* statistic. The prototype’s real-time seizure prediction and continuous operation of the wearable system will allow applications in the real world.

The MSPC model can be refined whilst it is operational via patient data updates. This could improve the model’s sensitivity and allow the model to adapt to shifts in a patient’s normal state.

The evaluation of the proposed system in the actual daily life of epilepsy patients is desired in future works.

## Figures and Tables

**Table 1:** Patient demographic and clinical characteristics. Medications were carbamazepine (CBZ), gabapentin (GBP), levetiracetam (LEV), valproic acid (VPA), rufinamide (RFN), lamotrigine (LTG), clonazepam (CZP), zonisamide (ZNS), and lacosamide (LCM).

Patient	Sex	Age	Seizure Foci	Medication (mg/day)
A	F	31	Right temporal lobe	CBZ 200, GBP 1200
B	M	54	Left mesial temporal lobe	LEV 500, VPA 1000
C	M	20	Left temporal lobe	LEV 1000
D	F	25	Undefined	RFN 600, LTG 150, LEV 2500, VPA 400
E	F	42	Occipital lobe (undefined lateralization)	LEV 2000, GBP 600, CZP 1, ZNS 300
F	M	9	Right frontal lobe	VPA 400, CBZ 200
G	F	14	Undefined	LEV 1750, LCM 50

**Table 2:** Collected episodes. Seizures were focal-impaired awareness seizures (FIAS), focal to bilateral tonic–clonic seizures (FBTCS), and focal aware seizures (FAS). “FIAS→FBTCS” means that FIAS symptomatically changed to FBTCS, which was treated as a consecutive seizure.

Patient	Seizures	Total Duration (h:min)	Interictal Duration (h:min)	Control (age/gender)	Total Duration (h:min)
A	3 FIAS	70:14	0:53	31/F	5:08
B	FIAS→FBTCS	40:35	13:47	57/M	4:48
C	2 FIAS	32:18	9:21	20/M	7:17
D	FIAS	105:52	2:57	25/F	7:08
E	2 FAS	85:03	2:43	45/F	11:21
F	2 FIAS	28:39	8:07	9/M	6:07
G	3 FAS	86:45	2:28	16/F	7:14

**Table 3:** Measured failure rate for all subjects.

Patient	Total RRIs	RRI Outliers	Failure Rate (%)
A	245,920	11,564	4.7
B	163,520	577	0.4
C	159,240	5805	3.6
D	474,630	31,665	6.7
E	343,770	14,668	4.3
F	144,530	2446	1.7
G	419,440	11,866	2.8

**Table 4:** Seizure prediction performance. The duration of prediction is shown with the start and the end of exceedance (e.g., −05:28 to −05:17) means that the statistic exceeded the control limit from 5 min 28 sec to 5 min 17 sec prior to a seizure. NA indicates not available, meaning that the statistic did not exceed the control limit. Sen (sensitivity) summarizes the true positive ratio of seizure prediction.

Seizure	Duration (min:s to min:s)	
	*Q*	*T* ^2^
**A1**	−05:10 to −04:58	NA
**A2**	−05:16 to −02:19	NA
**A3**	NA	NA
**B1**	−07:06 to −05:16	NA
**C1**	−14:40 to −14:25, −12:41 to −11:40	NA
**C2**	NA	−09:56 to −08:16
**D1**	−13:05 to −11:15	NA
**E1**	−16:09 to −14:44, −09:36 to −06:39	NA
**E2**	−14:43 to −10:36	NA
**F1**	−12:43 to −12:06	−13:18 to −10:28, −06:34 to −02:52
**F2**	−15:41 to −12:42, −10:13 to −08:44	NA
**G1**	−14:23 to −13:39, −08:37 to −04:54, −03:38 to −01:05	NA
**G2**	−03:52 to −02:59	NA
**G3**	−12:18 to −09:33	NA
**Sen**	85.7%	14.3%

**Table 5:** False positive rate during interictal period.

Patient	False Positive Rate (times/h)		False Positive Rate of Healthy Control	
	*Q*	*T* ^2^	*Q*	*T* ^2^
**A**	3.34	1.11	0	2.14
**B**	0.29	1.16	0	1.46
**C**	1.62	1.62	0.69	1.92
**D**	0.43	1.71	0.14	0.56
**E**	0.67	0.34	1.32	0.59
**F**	0.73	4.76	1.30	0.65
**G**	0.74	0.37	0.97	1.82
**Total**	0.62	1.34	0.93	1.02
